# Ultrasensitive quantification of PD-L1+ extracellular vesicles in melanoma patient plasma using a parallelized high throughput droplet digital assay†

**DOI:** 10.1039/d4lc00331d

**Published:** 2024-06-13

**Authors:** Hanfei Shen, Yasemin Atiyas, Zijian Yang, Andrew A. Lin, Jingbo Yang, Diao Liu, Juhwan Park, Wei Guo, David A. Issadore

**Affiliations:** a Department of Bioengineering, School of Engineering and Applied Science, University of Pennsylvania Philadelphia PA USA issadore@seas.upenn.edu; b Department of Radiology, School of Medicine, Stanford University Stanford California USA; c Department of Biology, School of Arts and Sciences, University of Pennsylvania Philadelphia PA USA; d Department of Electrical and Systems Engineering, School of Engineering and Applied Science, University of Pennsylvania Philadelphia PA USA

## Abstract

The expression of programmed death-ligand 1 (PD-L1) on extracellular vesicles (EVs) is an emerging biomarker for cancer, and has gained particular interest for its role mediating immunotherapy. However, precise quantification of PD-L1+ EVs in clinical samples remains challenging due to their sparse concentration and the enormity of the number of background EVs in human plasma, limiting applicability of conventional approaches. In this study, we develop a high-throughput droplet-based extracellular vesicle analysis (DEVA) assay for ultrasensitive quantification of EVs in plasma that are dual positive for both PD-L1 and tetraspanin (CD81) known to be expressed on EVs. We achieve a performance that significantly surpasses conventional approaches, demonstrating 360× enhancement in the limit of detection (LOD) and a 750× improvement in the limit of quantitation (LOQ) compared to conventional plate enzyme-linked immunoassay (ELISA). Underlying this performance is DEVA's high throughput analysis of individual EVs one at a time and the high specificity to targeted EVs *versus* background. We achieve a 0.006% false positive rate per droplet by leveraging avidity effects that arise from EVs having multiple copies of their target ligands on their surface. We use parallelized optofluidics to rapidly process 10 million droplets per minute, ∼100× greater than conventional approaches. A validation study on a cohort of 14 patients with melanoma confirms DEVA's ability to match conventional ELISA measurements with reduced plasma sample volume and without the need for prior EV purification. This proof-of-concept study demonstrates DEVA's potential for clinical utility to enhance prognosis as well as guide treatment for cancer.

## Abstract 2

We develop a high-throughput droplet-based extracellular vesicle analysis (DEVA) assay for ultrasensitive quantification of EVs in plasma that are positive for both PD-L1 and tetraspanins.

## Graphical abstract

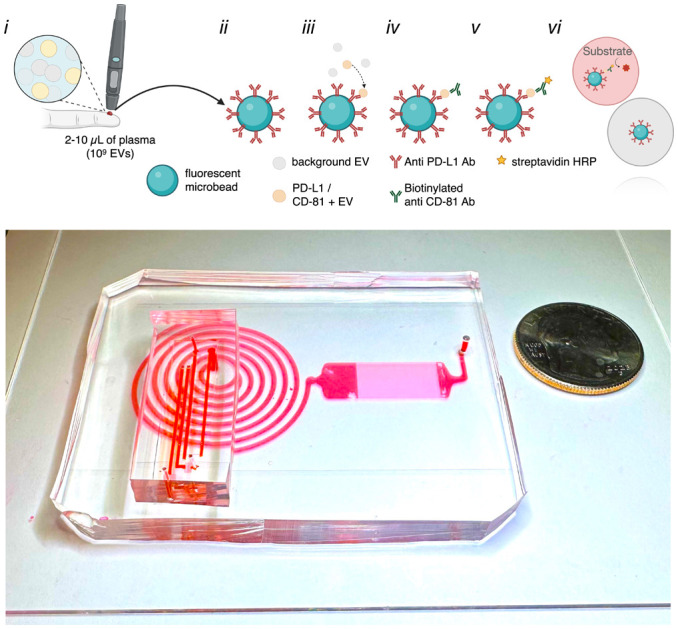

## Introduction

Extracellular vesicles (EVs) have attracted enormous attention for their diagnostic and therapeutic potential.^1–6^ The molecular cargo of proteins and RNAs packaged within EVs has been found to play important functional roles in many cancers, as well as a wide variety of other diseases.^2,7–9^ A key challenge in the study of the role of EVs *in vivo*, or for their application as biomarkers, is that EVs from diseased tissues are sparse in biological fluids, such as blood, which contain a background of ≈10^11^ EVs mL^−1^; in comparison, previous literature estimates that the concentration of tumor derived EVs of ≈10^3^ EVs mL^−1^ of blood per cubic mm of tumor volume.^10^ Moreover, the EVs of interest often contain similar molecules as the background EVs, with only nuanced differences in their cargo. Yet, the specific packaging of multiple molecular cargo into each individual vesicle is believed to be key to their functionality.^11–13^ Indeed, heterogeneity amongst individual EVs has become a central topic in EV biology as it is manifested in a wide range of experimental (*e.g.* cell culture) and pathophysiological contexts (*e.g.* plasma and tissues).^14–16^ The detection of rare, and often heterogeneous, target EVs amongst a background of an enormous number of heterogeneous EVs cannot be achieved using conventional, or even recently developed microfluidic approaches,^17–19^ which analyze EVs together in bulk; it must be addressed at the single EV level.

Recent advances in microfluidics have led to the development of technology platforms capable of sorting and characterizing single EVs. However, most commercial, and recently developed, single-particle EV measurement technologies are based on direct visualization of individual EVs,^20–22^ which suffer from the fundamental limitation that they can only analyze ≲10^4^ EVs, which is orders of magnitude fewer than found in clinical samples. Digital droplet microfluidics, where individual EVs are encapsulated in droplets for downstream analysis, has emerged as a promising solution for higher-throughput EV characterization.^23–25^ However, it has remained a challenge to achieve the necessary technological trifecta of high sensitivity to detect individual EVs, high specificity to identify specific EV subsets based on multiple surface proteins, and high throughput necessary for studying rare EV populations.^26^ Leveraging recent innovations in high-throughput droplet microfluidics, we recently developed a novel platform that performed ultrasensitive detection of individual EVs in complex biological backgrounds, which we coin droplet-based extracellular vesicle analysis (DEVA).^25^ In an initial proof-of-concept publication, we quantified EVs positive for the tetraspanin marker CD81. To evaluate the performance of DEVA, we used a model system of human cell culture-derived EVs spiked into a background of fetal bovine serum (FBS), and achieved a limit of detection (LOD) of 9 target EVs per μl, which was over a 100-fold improvement in LOD compared to gold-standard conventional ELISA.^25^

In this paper we build on our prior work to develop a DEVA-based assay for ultrasensitive detection of PD-L1+ EVs directly within human plasma from melanoma patients. Our focus on melanoma stems from the remarkable efficacy of immune checkpoint blockade (ICB)-based therapies for melanoma, such as the use of anti-PD-1 antibodies. Despite enormous progress, the majority of patients still fail to respond to treatment, highlighting the urgent need to understand the intricacies of tumor–immune system interactions that influence treatment outcomes.^27^ One particular focus in melanoma research is the role of PD-L1, a protein expressed on the surface of cancer cells and the EVs derived from them, in the process of immune evasion.^28,29^ Melanoma cells, especially metastatic ones, release EVs that carry PD-L1.^30–32^ PD-L1 molecules expressed on the cell surface interact with the PD-1 receptors highly expressed on T cells, and this interaction protects normal cells from an immune attack by signaling T cells to reduce their activity.^33,34^ However, many cancer cells, including melanoma cells, exploit this mechanism by overexpressing PD-L1 to effectively suppress the immune system, promoting the tumor growth and spread.^34^ Recent research has also highlighted the role of cancer-derived EVs in this process. These PD-L1+ EVs, when interacting with immune cells, can inhibit T cell function and induce immune tolerance, much like PD-L1 on the surface of cancer cells.^30,35,36^ This mechanism further contributes to the immune evasion capabilities of tumors. The understanding of PD-L1's role on cancer cells and their derived extracellular vesicles has been pivotal in prognosis and predicting outcomes of therapies targeting the PD-1/PD-L1 immune checkpoint.^29,30,37^ However, previous attempts to profile PD-L1+ EVs have been limited by technology that either analyzes bulk EV populations, such as plate ELISA, or technologies that can resolve individual EVs but that lack the throughput to sample enough EVs to reliably detect rare PD-L1+ EVs in blood.^20–22^

In this study, we develop a DEVA-based platform to detect EVs with ultrahigh sensitivity that are dual positive for PD-L1 and a tetraspanin, known to be enriched on exosomes and other small EVs,^38,39^ directly in plasma samples. Measuring PD-L1+/CD81+ dual positive EVs both allows for direct measurement of PD-L1+ EVs without EV purification, and also allows us to focus on exosomal PD-L1+ EVs, which were demonstrated to be an informative biomarker for diagnosis and immunotherapy outcome prediction of melanoma in previous work.^30^ By performing sandwich digital ELISA and leveraging avidity effects that arise from EVs having multiple copies of their target ligands on their surface, we can achieve a high specificity to targeted EVs *versus* background (0.006% false positive rate per droplet, and 0.06% false positive rate per bead). By performing this digital droplet assay at high throughput (>10^7^ droplets per minute) using our parallelized microfluidic chip, we sample enough droplets that even for very sparse EVs we are not limited by Poisson counting error.^25^ Using cell culture derived EVs, we achieved an LOD that is 360× lower and a limit of quantitation (LOQ) that is 750× lower than what is achieved using conventional plate ELISA in a head-to-head comparison using the same antibodies and cells. Using the same DEVA assay, we quantified EVs in a set of plasma samples from a cohort of subjects with melanoma (*n* = 14). We demonstrated that using a sample volume as small as 2 μL, our assay could reliably quantify dual PD-L1+/CD81+ EVs and match the results of conventional plate ELISA that used 100 μL of plasma. These proof-of-concept experiments demonstrate the potential of our high-throughput single EV technology to uncover novel tumor–immune system interactions mediated by EVs and paves the way for developing new EV biomarkers to better refine and personalize the diagnosis and treatment of cancer.

## Experimental

**Fig. 1:**
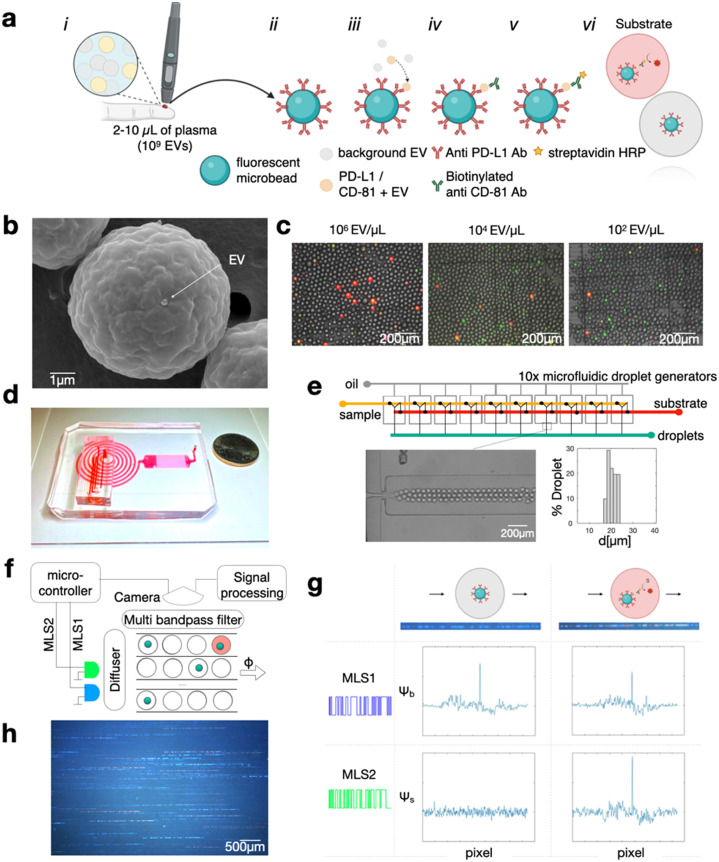
Overview of the high-throughput digital assay for quantifying PD-L1+/CD81+ EVs. (a) Diagram illustrating the process of PD-L1+/CD81+ EV quantification using the digital assay. Workflow depicting the generation of a digital signal from a small volume of blood (less than 10 μl of plasma). The workflow consists of (i) acquisition of plasma, (ii) capture of target EVs onto antibody functionalized microbeads, (iii) labeling of captured EVs with labeling antibody, (iv) tagging the labeling antibody with HRP enzyme, and finally (vi) digital encapsulation of beads into substrate filled droplets for fluorescence read-out. (b) Scanning Electron Microscopy (SEM) image demonstrating that typically no more than one EV is bound to each microbead. (c) Fluorescence microscopy image illustrating the digital signal obtained at various mel-B7H1 EV concentrations. (d) Image of the microfluidic chip designed for droplet generation, incubation, and video recording. (e) Illustration of a parallel droplet-generator system, which produces droplets averaging 20.4 μm in diameter with a coefficient of variation (CV) of 9.8%. (f) Conceptual diagram of signal generation in DEVA, highlighting the use of laser diodes to excite microbead and ELISA signals which are modified with maximum length sequences (MLS) to create patterned streaks as droplets with beads and/or positive ELISA signal move across the imaging area. (g) Bead and ELISA signals can be distinguished through correlation-based analysis, with coinciding bead and ELISA signals indicating dual PD-L1+/CD81+ EV. (h) Representative image showcasing the imaging area of DEVA.

We designed a droplet-based high throughput digital ELISA assay to quantify EVs that are dual-positive for the tetraspanin CD81 (confirmed in this study to be expressed on PD-L1+ EVs) and PD-L1, using a sandwich-based approach (Fig. 1a). In this workflow, any EV that is positive for PD-L1 is captured onto an anti-PD-L1 functionalized fluorescent microbead, with the number of beads large enough such that each bead captures either 1 or 0 EV (Fig. 1b, ESI† Fig. S1). EVs that are captured on the microbeads are subsequently labeled with biotinylated anti-CD81 labeling antibodies, which attach streptavidin-horseradish peroxidase (HRP) enzymes to any bead that has captured one of these targeted EVs. The beads are then mixed with HRP substrate and partitioned into droplets such that each droplet contains only 1 or 0 beads. Any droplet that contains HRP will fluoresce (Fig. 1c). Each droplet is interrogated using our optofluidic DEVA chip to count the ratio of beads that have captured the target EVs, reported as the average EVs per bead (AEVB). Due to high specificity of digital droplet sandwich ELISA, DEVA has a false positive rate per bead of only AEVB_b_ = 0.06% (a false positive rate per droplet AEVB_d_ = 0.006%), which is 1–2 orders of magnitude improved compared to competing approaches.^40–43^ This low false positivity motivates us to measure droplets at a high throughput such that we can quantify enough beads so we are not limited by Poisson counting error,^25^ resulting in >10^5^ beads per measurement, and >1 million drops per measurement. Achieving this throughput is enabled by the DEVA platform (Fig. 1d), which features parallelized microfluidic droplet generators and parallelized in-flow droplet time-domain encoded fluorescence imaging channels, which together are capable of processing >100 000 droplets per second.

Briefly, our DEVA workflow to quantify EVs that are dual positive for PD-L1 and CD81 is as follows. The sample is incubated for 12 hours at 4 °C with 10^6^ fluorescently labeled immunoaffinity beads (Sphero™ Fluorescent Carboxyl Magnetic Particles, Yellow, 5.65 μm, Spherotech) in a volume of 100 μL. If the sample volume is less than 100 μL, it is diluted using a custom buffer solution described below. The microbeads are functionalized with capture antibodies *via* carboxyl binding (PolyLink Protein Coupling Kit, Polysciences).

Subsequently, the beads are incubated for 1 hour at room temperature (RT) with 100 μL of 2 μg ml^−1^ concentration of biotinylated detection antibody to label the captured EVs. The formation of a bead–EV–enzyme complex is completed with a 15 min incubation with 100 μL of HRP-streptavidin (ThermoFisher) diluted at 1 : 10 000, which labels the detection antibodies that are bound to the captured EVs with biotin (Fig. 1a). For direct quantification of PD-L1+/CD81+ EVs from clinical samples in this study, antibodies targeting PD-L1 (clone 29E.2A3, Biolegend) and CD81 (clone REA513, Miltenyi) were selected as capture and detection antibodies, respectively. In isotype negative control experiments, Purified Mouse IgG2b, κ Isotype Ctrl Antibody (Biolegend) were used as capture antibody coated on the microbeads. The CD81 antibody underwent biotinylation *via* the One-Step Antibody Biotinylation Kit (Miltenyi), enabling the attachment of HRP-streptavidin. Subsequently, the beads are loaded into droplets that contain QuantaRed™ Enhanced Chemifluorescent HRP Substrate Kit (ThermoFisher). This substrate was used as the reaction substrate in both DEVA and conventional ELISA assays. We developed a custom buffer, comprising 75% v/v SuperBlock™ Blocking Buffer in PBS (ThermoFisher), 25% v/v PBS, and 1% w/v BSA, selected to maximize the performance of the DEVA assay.^25^ This buffer was used for both washing procedures and the dilution of antibodies and HRP-streptavidin for DEVA experiments.

We perform DEVA on an integrated microfluidic platform that generates, processes, and reads out the result of the assay at a throughput of 10^7^ droplets per minute (Fig. 1e). The droplet generation is performed using 10× flow focusing droplet generators connected in parallel on the same chip using a previously described ladder geometry, fabricated using double-side imprinted soft lithography.^25,44^ The droplet generator operates at a throughput of 10^7^ droplets per minute, generating aqueous droplets (QX200™ Droplet Generation Oil for EvaGreen, Biorad) that contain the bead–EV–enzyme complex and the ELISA substrate. Droplets that are produced have an average diameter of 20.4 μm in diameter with a coefficient of variation CV = 9.8% (Fig. 1e). The contents of the droplets are mixed immediately upstream of each droplet generator to avoid the HRP substrate reacting with HRP enzyme before the beads are encapsulated within their individual droplets, which otherwise could cause a falsely positive signal. Downstream of the array of droplet generators, we incorporate a large cross-sectional area channel (1980 (*w*) × 330 (*h*) μm^2^) designed to provide sufficient time for enzymes in the droplets to convert the fluorescent substrate (∼1 min). The processed droplets are then directed into a detection region, where they are evenly distributed across 90 parallel channels where their fluorescence is interrogated. In this detection region we use time domain modulated fluorescence detection^25,45^ to assess whether a droplet contains a bead, and whether that anti PD-L1 functionalized bead has captured an EV, which was labeled with an anti-CD81 antibody, which has resulted in the conversion of the fluorescent substrate (Fig. 1f).

The time domain modulated fluorescence detection scheme that we use to measure the results of our DEVA assay has been described in detail previously.^22,44^ Briefly, the excitation for fluorescence is provided by two time-domain-modulated laser modules (Techhood), one blue and one green, with wavelengths *λ*_exBlue_ = 457 nm and *λ*_exGreen_ = 528 nm. The emission of the passing droplets is measured over a field of view FOV = 15 mm × 9 mm using a digital camera (Grasshopper3, GS3-U3-23S6C-C: 2.3 MP). Each light source is modulated in time using a microcontroller that modulates each light source with a distinct maximum length sequence (MLS), selected such that each sequence has minimal autocorrelation and minimal cross-correlation with one another. The MLS pattern is 63 bits long and has a period matched to the exposure time of the camera. The camera has a global shutter and an exposure time of 45 ms. The modulation of the light sources at a rate greater than the frame rate of the camera allows the fluorescent bead and the fluorescent substrate in the droplet to be imaged as an MLS-encoded streak (Fig. 1f). The purpose of this MLS encoding is that it allows nearby droplets to be resolved by correlation-based analysis, even when their streaks overlap in space. Moreover, since the fluorescent beads and substrate have non-overlapping excitation spectra, they can be excited by different light sources, allowing their fluorescence emissions to be distinctly encoded by two different MLS patterns. We can thus distinctly quantify the fluorescence signal that corresponds to the fluorescent bead *versus* that which corresponds to the fluorescent substrate *via* correlation analysis (Fig. 1g). This strategy allows accurate detection of the bead and fluorescence substrate signal, across each of the 90 parallel channels simultaneously, resulting in a throughput of 10^7^ drops per minute.^45^ The AEVB is the total number of dual positive events, droplets that contain a bead and in which the substrate fluoresces and therefore has captured a targeted EV, divided by the total number of beads measured (Fig. 1h).

To detect PD-L1+ EVs using conventional plate ELISA, we adapted a protocol in the publication by Chen *et al.*,^30^ modifying the choice of reagents, including antibodies and enzymes, to align with those used in the DEVA assay. PD-L1 antibody (clone 29E.2A3, Biolegend) was applied to each well at a concentration of 5 μg mL^−1^ per well (50 μL) and allowed to coat overnight at 4 °C. Subsequently, each well was blocked with SuperBlock (in PBS) blocking buffer (ThermoFisher) for 2 hours at RT. Then, 100 μL of isolated EV samples, prepared using Total Exosome Isolation Kit (Invitrogen), were added to each well at various concentrations and incubated overnight at 4 °C. The detection antibody CD81 (clone REA513, Miltenyi) was added at a concentration of 1 μg mL^−1^ per well (100 μL) and incubated for 1 h at RT. Afterward, 100 μL of HRP-streptavidin (ThermoFisher), diluted 1 : 10 000 in SuperBlock blocking buffer, was added to each well and incubated for 15 minutes at RT before subject to measurements. During the sample incubation step, we directly coated recombinant CD81 (Abcam) at various concentrations (0.01–100 ng mL^−1^) onto empty wells to generate a standard curve. Fluorescent measurements were then conducted using a plate reader (Infinite M PLEX, TECAN). The washing buffer was prepared by diluting 0.5% Tween-20 (Sigma) in PBS. To quantify EV concentration in cell culture derived sample, we first performed purification of EV from cell culture media with Total Exosome Isolation Kit (from cell culture media) (Invitrogen). The isolated EV samples were serially diluted and measured by a ZetaView PMX220 Twin at the Extracellular Vesicle Core (School of Veterinary Medicine, University of Pennsylvania). Scanning electron microscope (SEM) imaging was conducted at the Cell and Developmental Biology Microscopy Core (Perelman School of Medicine, University of Pennsylvania).

The culture of two cell lines, a melanoma cell line 624-mel, and mel-B7H1, a cell line generated by Dong, *et al.*, *via* transfecting a 624mel with a B7-H1 expression vector^46^ were provided by H. Dong (Mayo Clinic). The cell lines are cultured within RPMI 1640 medium (Invitrogen) supplemented with 10% exosome-depleted FBS (Invitrogen) (exosome depleted *via* overnight centrifugation at 100 000*g*). The supernatants were collected from 48–72 h cell culture.^30^

Plasma sample collection was subject to a protocol identical to that reported in the previous paper by Chen *et al.*^30^ Patients provided written consent for blood collection as part of the University of Pennsylvania Abramson Cancer Center's melanoma research program tissue collection protocol UPCC 08607, in compliance with the ethics committee and Institutional Review Board of the University of Pennsylvania.

## Results and discussion

**Fig. 2:**
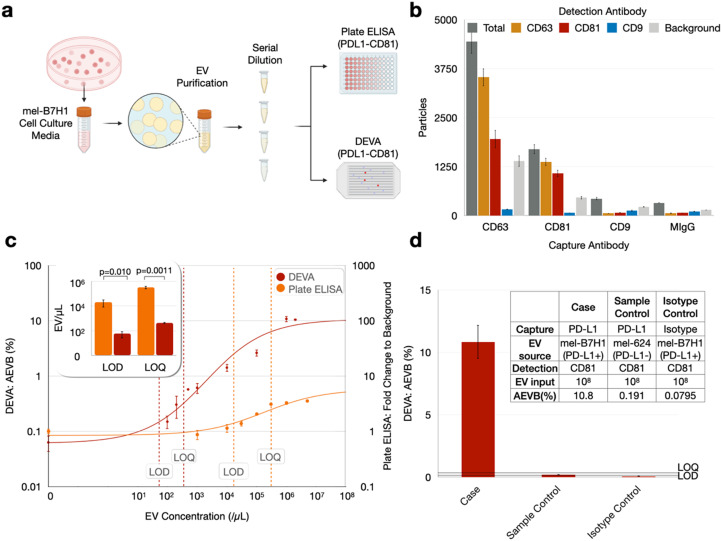
Comparative analysis of PD-L1+ EV quantification using DEVA *versus* conventional ELISA in *in vitro* samples: (a) methodology for establishing titration curves for both conventional sandwich ELISA and DEVA, utilizing cell culture media from the mel-B7H1 cell line. (b) Analysis of surface tetraspanin markers on mel-B7H1 EVs *via* ExoView, highlighting the absence of CD9 expression and identifying CD81 as having the superior signal-to-background ratio (*n* = 3 replicates). Light gray bars indicate background EVs detected by ExoView's interferometric reflectance imaging sensor but not labeled with any fluorescent detection antibody. (c) Titration curves for DEVA and conventional ELISA, demonstrating DEVA's significantly enhanced LOD and LOQ (*n* = 3 replicates for DEVA's blank sample, *n* = 2 otherwise). (d) Sample control using mel-624 EVs and isotype controls with non-specific capture antibodies at high EV input concentrations on DEVA maintained low background levels below the LOD (*n* = 2 replicates). All error bars represent the standard error of the mean.

We first evaluated DEVA's capability to quantify PD-L1+/CD81+ EVs using a cell culture model for human cancer cell derived EVs that are positive for PD-L1 (Fig. 2a). We used the human cell model mel-B7H1, which is engineered to overexpress PD-L1 on the cell surface, as the source of target EVs. We first characterized mel-B7H1 EVs using gold standard EV analysis technologies. Using nanoparticle tracking analysis (NTA) we found the concentration of EV sized (30–1000 nm) particles *C* = 1 × 10^7^ μL^−1^ (ESI† Fig. S2). Subsequently, we profiled the expression of tetraspanin surface markers (CD9, CD63, and CD81) on the mel-B7H1 derived EVs using ExoView (NanoView) assay, and found low CD9 expression and higher levels of CD63 and CD81 (Fig. 2b). Based on this result, we chose to use CD81 in our DEVA assay because it had the greatest ratio between signal and non-specific background as measured using ExoView's interferometric reflectance imaging sensor.

**Fig. 3:**
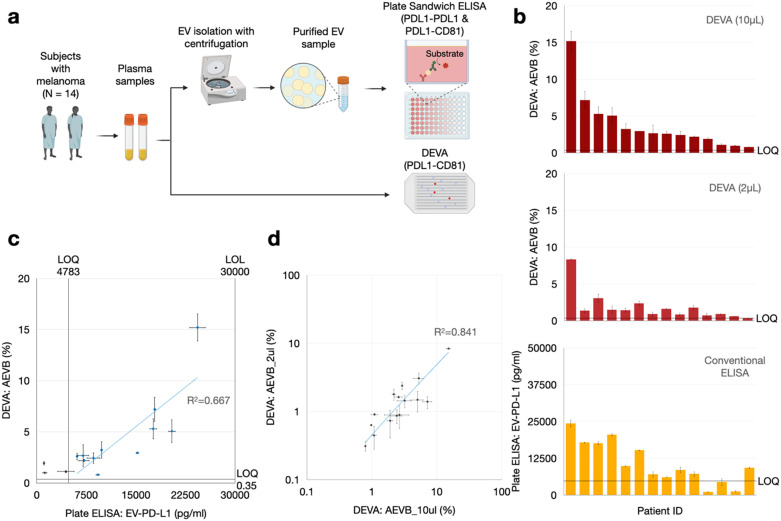
Comparative evaluation of PD-L1+ EV quantification in clinical samples using DEVA and conventional ELISA: (a) procedures employed for quantifying PD-L1+ EVs from patient plasma samples using both conventional sandwich ELISA and DEVA. (b) Histogram showing the signal for all samples measured by DEVA (10 μL and 2 μL input) and conventional ELISA relative to their corresponding LOQ, indicating DEVA's enhanced sensitivity. (c) Comparison of signal measured with the two assays indicate positive correlation. Blue data points indicate the samples for which both DEVA (10 μL) and conventional ELISA measurements cleared their corresponding LOQ. (d) DEVA's 10 μl and 2 μl input signals on the patient samples demonstrated positive correlation. All error bars represent the standard error of the mean for *n* = 2 replicates.

We chose to use PD-L1 as the capture antibody and CD81 as the detection antibody for several reasons. First, we found *via* plate ELISA that even for mel-B7H1-derived EVs, which are known to express PD-L1,^30–32,47^ there was greater labeling of CD81 than PD-L1 on the EVs (ESI† Fig. 3). Thus, we anticipated an improved avidity for CD81 antibody labeling compared to what we would have with PD-L1, which improves the specificity of the assay.^25^ Second, if we were to use CD81 as the capture antibody, we would need a much greater number of beads than what is required using PD-L1 as a capture antibody to achieve digital loading of EVs onto the microbeads. Because tetraspanins are expressed across many types of EVs in blood, we would need enough beads to capture ∼10^11^ EVs mL^−1^ of plasma.

We subsequently quantified the performance of DEVA using these cell culture model derived EVs and benchmarked it using a head-to-head comparison with conventional plate ELISA. The cell culture media was harvested following incubation with mel-B7H1 cells and subjected to EV isolation using the Total Exosome Isolation kit (from cell culture media) (Invitrogen). Using these isolated EVs resuspended in PBS, we formed a dilution series of EVs, with concentrations ranging from 10^2^ EVs μL^−1^ to 2 × 10^6^ EVs μL^−1^, to quantify and benchmark the performance of DEVA against conventional plate ELISA (Fig. 2a). Blank samples, which contained zero target EVs, were also measured using both technologies. By measuring this dilution series and blank samples in duplicate, for both technologies, we first determined the mean background level as AEVB_b_ for DEVA and fold change to background for plate ELISA, plus their 3 and 10 standard deviations. Next, we calculated the input concentration of EVs that corresponded to these quantities on our dilution series to determine the LOD and LOQ, respectively, as EV count per μl. For plate ELISA we found an LOD = 2.05 × 10^4^ EV μL^−1^ and LOQ = 3.24 × 10^5^ EV μL^−1^. For DEVA we found a dramatically improved LOD = 57.0 EV μL^−1^ and LOQ = 434 EV μL^−1^, a 360× improvement for LOD and 750× improvement for LOQ relative to plate ELISA (Fig. 2c). This enhancement in performance agrees well with existing literatures that compared the limits of detection (LOD) of digital assays against that of conventional ELISA techniques.^48–50^ We chose plate ELISA to compare the performance of DEVA because it is the gold standard protein quantification technology, and because plate ELISA-based assays have previously been established to measure exosomal PD-L1.^30^ However, the two technologies measure related but different quantities. While DEVA counts dual PD-L1+/CD81+ EVs, plate ELISA measures total EV PD-L1 protein. These two quantities differ because EVs are heterogeneous and have diverse amounts of PD-L1 on their surface. We therefore expect their results to be related but not identical, which is a limitation of this study. As protein-specific single EV detection technologies become more established, they can serve as a better tool to benchmark our assay in future studies.

To validate our measurements, we compared the results of DEVA with two negative controls. We repeated the DEVA experiments described above using an isotype control antibody (replacing capture antibodies). We also repeated the DEVA experiments described above using 624mel derived EVs (a cell line lacking PD-L1 expression). For both controls, using our highest concentration of target EVs 10^6^ EVs μL^−1^, we recorded a negative signal below our LOQ, AEVB = 0.08% and 0.19% respectively (Fig. 2d). In each of the DEVA experiments described, we measure ∼10^6^ droplets, chosen to be sufficiently large that the number of positive signals quantified can be measured without being dominated by Poisson counting error. While we aim to minimize bead aggregation by adding BSA and SuperBlock to the assay buffer, it is difficult to prevent it completely (ESI† Fig. S5). Because we use an excess of beads in each assay (10^6^ beads compared to the ∼10^5^ that we quantify), the ∼20% reduction in total bead number due to aggregation does not have a meaningful effect on the performance of our assay. We note that the presence of EVs does not significantly affect bead aggregation compared to the aggregation found with beads only.

We performed a head-to-head comparison of DEVA *versus* conventional plate-based ELISA in quantifying PD-L1+/CD81+ EVs from a set of *n* = 14 plasma samples from patients with melanoma. We did this comparison using a cohort of patients large enough to statistically compare our results *versus* that of a gold standard method, and to lay the groundwork for a much larger set of clinical measurements to validate the clinical utility of our technology. For each patient, we first quantified the number of dual PD-L1+/CD81+ EVs using 100 μL of sample, from which we isolated EVs with Total EV Isolation Kit (from plasma) (Invitrogen) and performed plate ELISA, which we compared to DEVA. We measured the same samples with DEVA using only 10 μL and 2 μL of volume of the unprocessed plasma. For conventional ELISA, 100 μL of plasma underwent total EV isolation using the Total Exosome Isolation Kit (Invitrogen), was diluted 1 : 5, and subjected to conventional sandwich ELISA using the same antibodies as in the DEVA measurements (Fig. 3a and b). Additionally, to compare our measurements against an established assay, wherein circulating exosomal PD-L1 is utilized to differentiate responders from non-responders in melanoma immunotherapy,^30^ we conducted another conventional sandwich ELISA on a subset of sample (*n* = 9) due to the limitation of sample availability. In this assay, PD-L1 was employed both as the capture antibody (using clone 3F9) and the detection antibody (using clone 6G8).

We found good agreement between DEVA's measurements of 10 μL of plasma and the measurement of 100 μL of plasma using conventional ELISA using the identical antibody pair (PD-L1 and CD81). The correlation coefficient was 0.83 and *R*-squared value *R*^2^ = 0.67 (Fig. 3c). We also found a somewhat weaker agreement between DEVA's measurement on 10 μL of plasma with conventional plate ELISA when PD-L1 served as both the capture and detection antibodies,^30^ yielding a correlation coefficient of 0.76 and an *R*^2^ = 0.59 (ESI† Fig. S4). This moderate correlation was anticipated, given the difference in antibodies used across the two assays. These results collectively demonstrate a general agreement between DEVA and conventional ELISA measurements. The observed discrepancies between DEVA and conventional ELISA can be attributed to several factors. Primarily, DEVA's digital nature offers enhanced sensitivity and specificity, enabling it to resolve signals that fall outside the dynamic range of conventional ELISA. Additionally, the difference in the fundamental nature of the assays can contribute to this discrepancy: DEVA quantifies EV numbers with co-expression of PD-L1 and CD81, whereas conventional ELISA measures total surface antigens.

Our findings also confirmed that the ultrasensitivity of DEVA can be leveraged to quantify dual PD-L1+/CD81+ EVs in our pilot set of melanoma patients in volumes as small as 2 μL. For all patient samples, measurements derived from the 10 μl plasma samples consistently yielded measured PD-L1+/CD81+ EV concentrations AEVB that surpassed our LOQ. And, for 13 out of 14 of the DEVA measurements on the 2 μL samples, we measured PD-L1+/CD81+ EV concentration AEVB that exceeded our LOQ (Fig. 3b). Furthermore, a positive correlation was observed between the AEVB values from both the 10 μL and 2 μL inputs (Fig. 3d). We additionally performed a negative control using the patient samples, wherein we performed DEVA on a 10 μl aliquot from a plasma sample, which had the highest positive signal (AEVB = 7.19%) within the digital regime (<10%) and measured it using an isotype capture antibody, which resulted in a signal AEVB = 0.10%, below the LOD and LOQ of DEVA.

## Conclusions

DEVA's superior sensitivity and specificity, and its capability to detect targeted EVs directly in unprocessed plasma, make it a valuable tool to improve the quantifications of circulating PD-L1+ EVs for applications both as a clinical biomarker and for basic biology. The observed agreement between the DEVA, using volumes as small as 2 μL, and conventional ELISA offers robust evidence supporting the viability of integrating DEVA into clinical practice such as melanoma immunotherapy response prediction. The small volume requirements can be useful in longitudinal measurements, wherein sample collection using a finger prick can potentially be employed. A limitation of our approach is that due to its digital nature, it cannot quantify the amount of a surface protein on each EV, as has been attempted in related work.^40^ Moreover, compared to technologies such as surface plasmon resonance (SPR), which offers rapid, label-free, and quantitative profiling of EV proteins,^51,52^ and can be used to profile both the surface and cargo proteins of single EVs,^53,54^ our current technology cannot measure the expression levels of EV surface proteins or access intravesicular proteins. Additionally, compared to surface-enhanced Raman spectroscopy (SERS), which generates distinct signatures across EV subtypes both in bulk^55^ and at the single EV level,^56^ this first version of the work measures a single EV subpopulation. The main advantage of our technology over these alternatives is its high throughput, ultrasensitivity and specificity,^25^ which enables the detection of sparse EVs directly from unprocessed plasma among a huge amount of background EVs with an enhanced dynamic range of 57–10^6^ EVs μl^−1^. In future work, there is an opportunity to adapt our high throughput DEVA technology to also quantify the amount of protein on individual EVs. Additionally, in future work, we envision using the capability of DEVA for multiplexing^57^ to profile multiple sub-populations of PD-L1+ EVs from various sources of cells, such as tumor cells, tumor associated macrophages, and CD9+ T cells. To achieve this goal, DEVA can be adapted by replacing the tetraspanin antibody in our assay with surface markers specific for these particular cell types. Recent studies have indicated that, in addition to tumor cells and T cells, other cells such as those from the monocyte–macrophage lineage are critical components of the cancer tumor microenvironment (TME).^17,58^ The accurate measurement of these EV sub-populations derived from the various relevant cell types in clinical specimens can enhance our understanding of the complex tumor ecosystem, and play an important role for developing next generation strategies for both biomarker development and effective cancer treatment.

## Author contributions

The manuscript was written through contributions of all authors. All authors have given approval to the final version of the manuscript.

## Conflicts of interest

None.

## Supplementary Material

LC-024-D4LC00331D-s001
