# Supplementary material for: Ultrasensitive quantification of PD-L1+ extracellular vesicles in melanoma patient plasma using a parallelized high throughput droplet digital assay
Source: Lab Chip. 2024 Jun 13;24(14):3403–11. doi: 10.1039/d4lc00331d (PMC11235413; doi:10.1039/d4lc00331d)
Supplement: LC-024-D4LC00331D-s001 [file LC-024-D4LC00331D-s001.pdf]

Electronic Supplementary Material (ESI) for Lab on a Chip.
This journal is © The Royal Society of Chemistry 2024

Supplementary Information

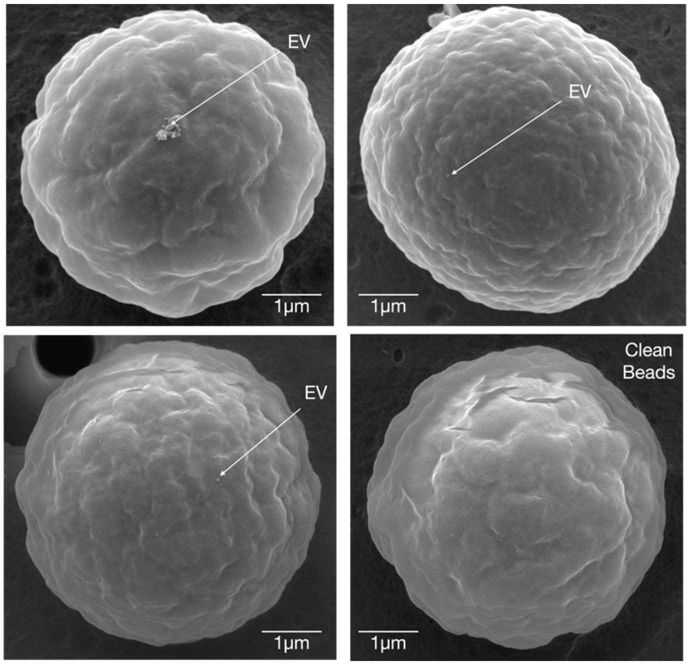

SI Figure 1. Additional SEM figures indicating one-to-one bead-EV binding, and a clean bead as a reference.

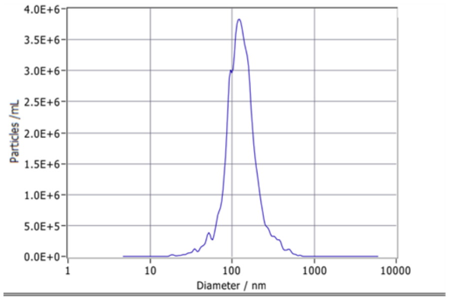

Peak Analysis (Concentration)

Diameter / nm	Particles/mL	FWHM / nm	Percentage
122.2	3.8E+6	96.2	96.6
52.2	3.8E+5	8.2	3.4

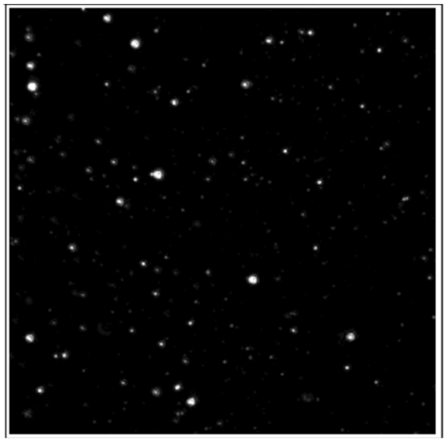

SI Figure 2. Nanoparticle Tracking Analysis of mel-B7H1 cell culture-derived EVs, purified with Total Exosome Isolation Kit (from cell culture media) (Invitrogen).

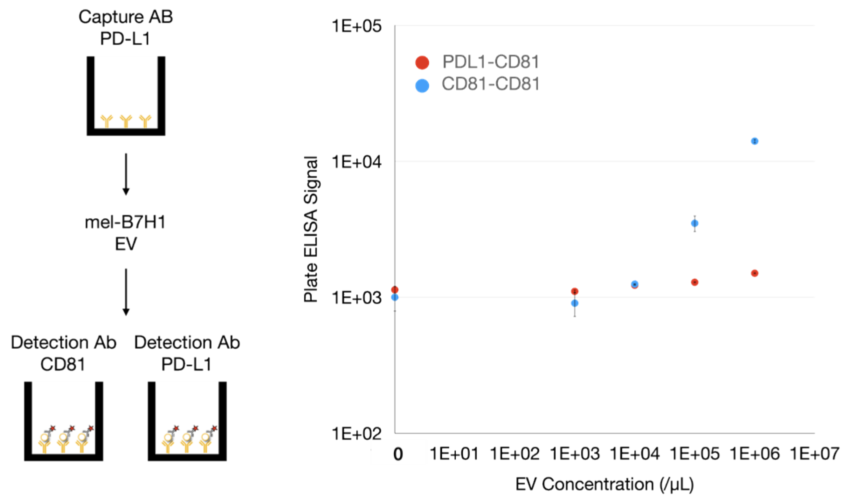

SI Figure 3. Plate-based sandwich ELISA demonstrated greater amount of mel-B7H1 cell culture-derived EVs express CD81 than PD-L1 on surface.

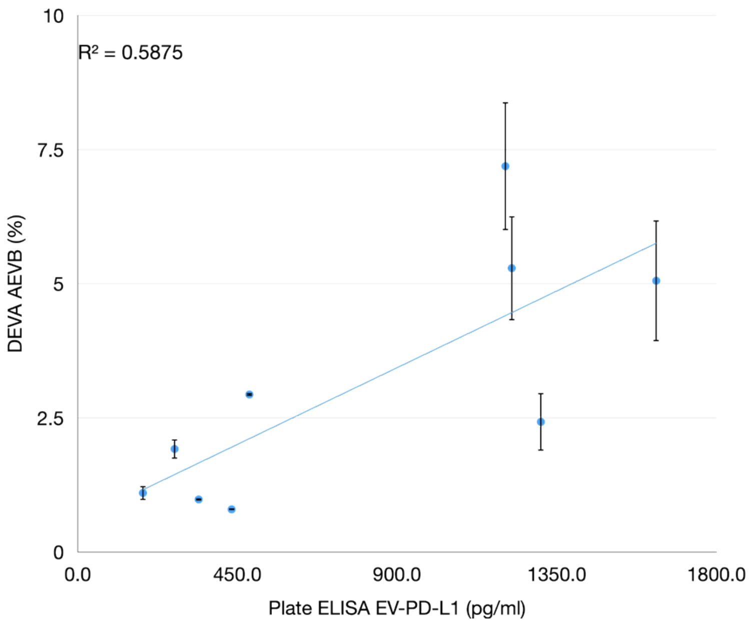

SI Figure 4. Comparing the signals of DEVA (10µL plasma) and plate sandwich ELISA using 9 patient plasma samples demonstrated positive correlation, with a Pearson correlation coefficient of 0.76 and an R^2 = 0.59

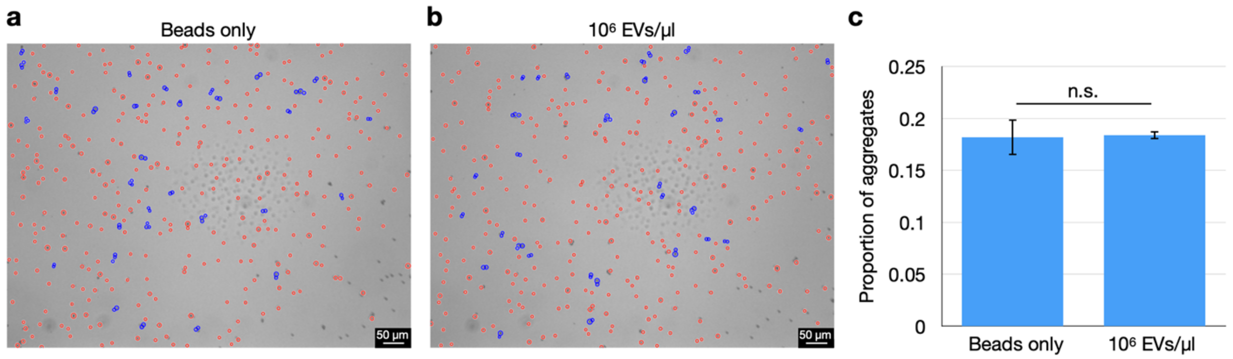

SI Figure 5. Quantification of bead aggregation due to magnetic washing or EV multivalency. Micrographs of the antibody functionalized beads used in our assay (*d* = 5.65μm, Spherotech) after being incubated alone (a) or co-incubated with 10^6 EVs/μl (b) in the assay buffer overnight, washed 2x using the magnetic stand, and resuspended in 100 μl assay buffer. Red circles label singlets while blue circles label aggregates. (c) The proportion of aggregates compared to single beads remain unchanged with the addition of EVs (N=1400-1800 beads per measurement).
